# Piloting a mental health intervention for young adults in poverty enrolled in post-secondary education in post-conflict regions in Colombia: a study protocol

**DOI:** 10.3389/fpsyt.2023.1238725

**Published:** 2023-11-13

**Authors:** Annie Zimmerman, María Camila García Durán, Ricardo Araya, Mauricio Avendano, Philipp Hessel, Yadira Díaz, Omar Dario Peña Niño, Sara Donetto, Martha Escobar Lux, Fabio Idrobo

**Affiliations:** ^1^Global Health and Social Medicine, King's College London, London, United Kingdom; ^2^Eje de Salud Poblacional, Fundación Santa Fe de Bogotá, Bogotá, Colombia; ^3^Health Service and Population Research Department, King's College London Institute of Psychiatry Psychology and Neuroscience, London, United Kingdom; ^4^Health Policy Unit at the Department of Epidemiology and Health Systems in Unisanté at the University of Lausanne, Lausanne, Switzerland; ^5^Escuela de Gobierno Alberto Lleras Camargo, Universidad de los Andes, Bogotá, Colombia; ^6^Florence Nightingale Faculty of Nursing, Midwifery and Palliative Care at King’s College London, London, United Kingdom; ^7^Departamento de Salud Mental, Fundación Santa Fe de Bogotá, Bogotá, Colombia; ^8^Facultad de Medicina, Universidad de Los Andes, Bogotá, Colombia; ^9^Department of Psychological and Brain Sciences, Boston University, Boston, MA, United States

**Keywords:** mental health, intervention, digital, trauma, young people, poverty

## Abstract

**Background:**

Colombia has endured more than five decades of internal armed conflict, which led to substantial costs for human capital and mental health. There is currently little evidence about the impact of incorporating a mental health intervention within an existing public cash transfer program to address poverty, and this project aims to develop and pilot a mental health support intervention embedded within the human capital program to achieve better outcomes among beneficiaries, especially those displaced by conflict and the most socioeconomically vulnerable.

**Methods:**

The study will consist of three phases: semi-structured one-to-one interviews, co-design and adaptations of the proposed intervention with participants and pilot of the digital intervention based on cognitive behavioral therapy and transdiagnostic techniques to determine its feasibility, acceptability, efficacy, and usefulness in ‘real settings’. Results will inform if the intervention improves clinical, educational and employment prospects among those who use it.

**Results:**

Knowledge will be generated on whether the mental health intervention could potentially improve young people’s mental health and human capital in conflict-affected areas? We will evaluate of the impact of potential mental health improvements on human capital outcomes, including educational and employment outcomes.

**Conclusion:**

Findings will help to make conclusions about the feasibility and acceptability of the intervention, and it will assess its effectiveness to improve the mental health and human capital outcomes of beneficiaries. This will enable the identification of strategies to address mental health problems among socioeconomically vulnerable young people that can be adapted to different contexts in in low and middle-income countries.

## Introduction

Internal conflicts lead to substantial human and economic losses for civilian populations (1). Physical assets are often destroyed or seized, and armed groups disrupt markets, seeking territorial strong-holds. Internal conflicts leave a legacy of structural poverty (2) and undermine young people’s development of human capital – defined as skills, knowledge, and experience necessary to succeed economically and improve their future. Human capital costs of armed conflict persist long after peace agreements have been reached (3). In turn, armed conflicts also have severe mental health consequences: young people exposed to conflict experience losses of relatives and friends, witness violent events, or can be the direct victims of violence, increasing the risk of mental health problems (MHP), such as post-traumatic stress disorder (PTSD), depression, and anxiety (4). MHPs can affect young people’s ability to build human capital and increase the risk of structural poverty, undermining peacebuilding efforts and restoring the vicious cycle.

### Mental health interventions for conflict victims

Colombia endured more than five decades of internal armed conflict, leaving high rates of MHP among Colombian youth between the ages of 13 and 28 (5). Epidemiological studies in conflict-affected areas have shown high prevalence rates of anxiety (52%), depression (43%), and PTSD (11–47%) (6–8), which often intersect with socioeconomic deprivation. Mental health interventions can help young people overcome the psychological effects of war and conflict, build resilience, and increase their ability to engage and succeed in their educational and training program (9), thus reducing the future risk of poverty. Our project aligns with recently enacted legislation in Colombia that aims to expand mental healthcare provision for the population affected by the internal conflict. This legislation led to the creation of the “Program of Psychosocial Care and Integral Health for Victims” (PAPSIVI) (10), together with a newly adopted National Mental Health Policy (11). Yet, evidence suggests that mental health services remain inadequate and underfunded, especially for young people (12). Less than 10% of the 9.3 million people recognized as conflict victims in Colombia have received some form of psychosocial support (13), and a treatment gap remains, with poor access to mental health services, particularly for those displaced by the conflict and more severe and complex problems that require specialist treatment (14).

### Digital mental health interventions and young people’s human capital

**Figure 1:**
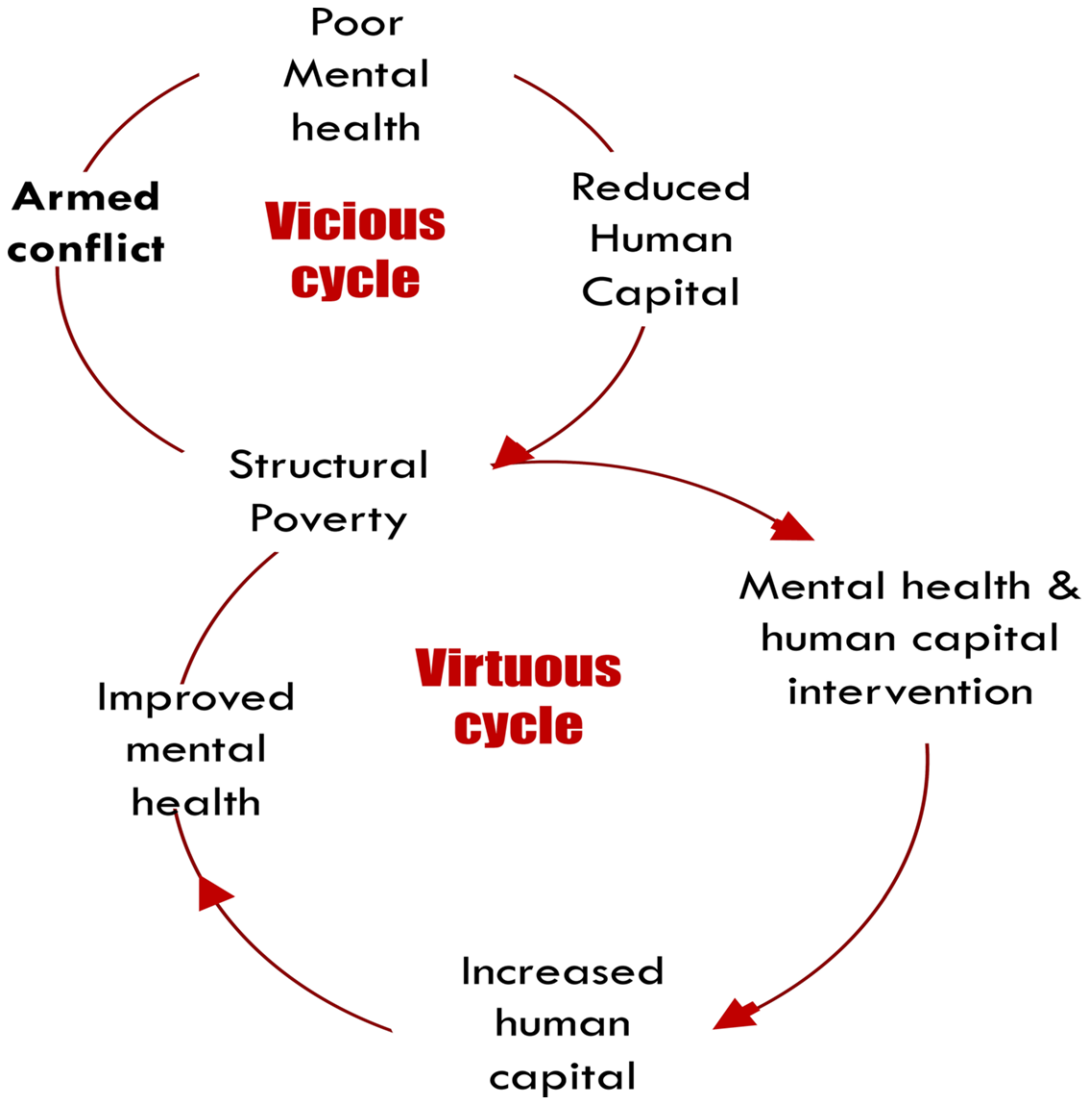
The vicious cycle of poverty, armed conflict, and mental health in low-and-middle-income countries (17).

Mental health is essential to human capital formation, influencing educational attainments and labor productivity (15, 16). Human capital and mental health can thus be part of a vicious cycle that limit young people’s ability to escape poverty in post-conflict societies. This project aims to demonstrate that jointly strengthening human capital (through the Youth in Action program) and improving mental well-being (through our intervention), can improve young people’s chances to enhance their learning outcomes and potentially interrupt the vicious cycle of poor attainment, poverty, and mental health problems. The combination of poverty and mental health problems (MHP) makes conflict-affected areas in Colombia a clear example of a vicious cycle (Figure 1). This project examines whether this vicious cycle can be broken through a mental health intervention embedded within a national cash transfer program that supports young people in accessing post-secondary education.

Our study builds upon evidence that training and education interventions with mental health programs substantially increase the likelihood of breaking the “vicious cycle” of poverty and poor mental health in young adulthood (18). There is some evidence that mental health interventions can help young people affected by conflict, but significant gaps in knowledge, with a lack of research evaluating the efficacy and feasibility of interventions (13). Some mental health interventions have shown partial success (13, 14). Still, high levels of mental comorbidity, low uptake and adherence, high dropout rates, lack of flexibility and trained therapists, and difficulties accessing treatment remain critical challenges.

### ‘Youth in Action’ program

The ‘Youth in Action’ program (Jóvenes en Acción in Spanish-JeA) is a major initiative of the Colombian government to decrease economic disparities by strengthening the formation of human capital among vulnerable young people living in poverty. Introduced in 2012, JeA supports young beneficiaries undertaking vocational training or university education with tuition fees and a living allowance given upon successful attendance and performance verification. JeA also offers a “skills for life” (Habilidades Para la Vida, in Spanish) training component to strengthen general skills that might increase long-term employability. The program currently hosts 445,000 beneficiaries, 14% living in conflict-affected municipalities. Although the program has been shown to impact educational outcomes and employment opportunities significantly, high levels of MHP among beneficiaries have been identified as an essential barrier to success (14).

### Digital mental health interventions in Colombia

There are few studies on the feasibility and impact of digital mental health interventions in Colombia. A recent study of an internet intervention for college students, assisted by a trained person, showed positive results but high dropout rates (around 80%) (19). Members of our team have developed and tested several simple mental health digital tools with good adherence and outcomes in other Latin American countries (20–23). With the advent of the COVID-19 epidemic, remote delivery is an additional underexplored challenge that this project aims to address. This study is particularly unique in that we work directly with Government within a public program. So far, no studies have assessed the impact of incorporating a mental health intervention within an existing public cash transfer program to address poverty.

## The present study

This study aims to design and pilot a mental health support intervention embedded within the JeA human capital program to achieve better outcomes among beneficiaries, especially those displaced by conflict and the most socioeconomically vulnerable. We aim to develop and test the feasibility and acceptability of a mental health intervention that addresses comorbid mental health problems among beneficiaries of the JeA program. We also aim to make a first evaluation of the impact of potential mental health improvements on human capital outcomes, including educational and employment outcomes.

We will also assess the several outcomes of the mental health intervention to improve the chances of JeA beneficiaries of completing the program and building human capital. Specifically, we will assess preliminary clinical outcomes of the relationship between the mental health intervention, compared to a control group, with dimensions related to human capital formation using a combination of administrative data and survey data collected as part of the intervention, that includes data on academic performance and attendance, as well as data on employment following the completion of the program. We also assess cross-site comparisons in efficacy to see if the effect differs by region. An analysis of the most recent national mental health survey data for conflict victims has not highlighted regional differences in the prevalence of common mental health disorders for victims of the conflict (7). They underscore the need for increased mental health services for this population in their regions. Notwithstanding, we will include in our statistical analysis tests for regional differences, comparing against control groups.

The project has three main research questions leading to methodological, empirical, and policy-related contributions:

1. Can a stepped-care mental health with a digital component improve young people’s mental health and human capital in conflict-affected areas?
2. Can this stepped-care mental health intervention improve young people’s chances of succeeding in their training and educational outcomes?
3. Is a stepped-care mental health with a digital component feasible and acceptable for inclusion in the JeA program in conflict-affected areas in Colombia?

We expect to address these aims in a period of 36 months (3 years).

### Methods and analysis

This study has three phases:

#### Phase 1 (6 months)

Phase 1 aims to determine the mental health needs and perceptions of the participants and facilitators of JeA and the barriers and facilitators that may contribute to the sustainability of the mental health intervention within the program. This will be done through literature and database review, a pre-pilot survey to study the prevalence of common mental health disorders in our population, and interviews.

##### Pre-pilot survey

We will send a pre-pilot survey to at least 50 participants of each territory to explore (a) the prevalence of common mental health disorders, specifically depression, anxiety, and post-traumatic stress disorder, and to (b) identify potential problems before implementing the full survey. We will include the same instruments and questions we intend to use in the survey on Phase 3 and add some feedback questions at the end to examine how clear the survey was and if there are any changes we need to consider. Participants will be asked for their consent before taking the survey and will be asked if they would also be willing to participate in qualitative activities.

##### Interviews

We will conduct formative research interviews to understand context but not to assess impact. A guide to interview participants and “Youth in Action” staff has been developed based on field work conducted in previous projects, where several of the authors participated, that studied the relation between mental health and poverty (24). The guide seeks to find the mental health resources that are available to our population and disposition to seek or recommend seeking help their willingness to participate in a mental health program. Participants and staffers will be encouraged to discuss these themes presented in the Appendix 1. The core of the discussion will be formative research that focuses on their understanding of mental health access to mental health services, and barriers/facilitators to access a mental health intervention. Questions will focus on ways to overcome stigma and recommendations on forms to generate acceptability of language related to mental health themes that will impinge on the willingness to answer our surveys. The invitations to participate in the intervention where we plan to outline the stepped care program will be co-written by JeA youth and staff to optimize engagement.

We will conduct semi-structured one-to-one interviews with 8 JeA staff to explore their experiences supporting young people with mental health issues and their views on potential barriers and facilitators to embedding the intervention in the JeA program. We will also carry 10 one-to-one interviews with young people who are beneficiaries of the JeA program to explore their views around mental health and their experiences of seeking or providing (to friends, for example) support for emotional distress and mental well-being. We will include young people enrolled in the JeA program, which will include youth with different characteristics, e.g., displaced people, victims of the armed conflict, indigenous people, and some who do not belong to any of these groups. The recruitment of these participants will be done by the regional staff of Youth in Action. They will provide us with a list of potential beneficiaries who are interested on participating and the research team will randomly select ten of them.

Once the intervention is finalized, we will also re-engage with participants to further understand the perceptions of usability, engagement, and acceptability of our program. In this initial stage, we also intend to prepare for difficulties with technology in the population we plan to engage. We have anticipated, using our most recent post-COVID national mental-health surveys, what the most present needs of this population might be. Our surveys will not be used to categorize participants to their assigned strategy but will help us complement these findings for conflict zones to potentially suggest policy initiatives to the governmental agency involved in this project.

The qualitative interviews will be recorded via Teams in video or audio, according to the participants’ preferences. Trained research team members will carry them out after informed consent is signed and before administering the questionnaires and starting the intervention. Recordings will be de-identified and kept on safe online servers.

#### Phase 2 (12 months)

Phase 2 consists of the co-design and adaptations of the proposed intervention with JeA participants. Currently, the program does not have a mental health program that caters specifically to its beneficiaries. To design the intervention, the research team will invite JeA beneficiaries and staff to three group discussions led by a facilitator and based on co-design principles and practices. Participants will be recruited using a list of beneficiaries who stated their interest in participating in qualitative research through the pre-pilot survey sent in Phase 1 Furthermore, participants who were interviewed in Phase 1 will also be invited to participate in these interactions. These sessions will enable participants to share their perspectives on mental health issues for young people in Colombia and then discuss and refine the essential features of the intervention and its delivery strategies. The guiding questions to elicit opinions and points of view will focus on the acceptability of a digital platform and encourage the use of mental health platforms available on a trial basis (Appendix 2). If no changes are possible on the digital intervention, we will listen to young people’s views about how this platform could be better delivered and use this information to adapt our intervention framework. The refined intervention will then be presented to JeA beneficiaries during a final co-design meeting to identify any more necessary adaptations. In this phase, we will also pilot the survey from which we will obtain the data.

##### Intervention development

While the digital intervention was already established, the role out and launch of the study was developed in workshops and formative interviews. Previous interventions have shown that digital initiatives have a high dropout rate and are more effective when guided by coaches. To anticipate this possibility, we will hold a virtual workshop with stakeholders (six JeA central staff and four from the FSFB) to draw a joint Theory of Change plan and a clear plan of action to launch the pilot study (Phase 3). The main stakeholders involved in the intervention development are JeA beneficiaries and staff. We will aim to conduct this work in small groups of between 4 and 8 people for staff-only and beneficiaries-only discussions and 10–15 people for joint workshops, representing all the territories involved. Team members with experience in facilitating will plan each group session. These team members will also facilitate the workshops. Potential participants will be identified and recruited with the help of JeA staff members.

#### Phase 3 (18 months)

During Phase 3, we will pilot the intervention to determine its feasibility, acceptability, efficacy, and usefulness in ‘real settings’. Results will inform if the intervention improves clinical, educational and employment prospects among those who use it. A sample of beneficiaries studying in our areas of interest will be invited to participate in a survey containing measures of mental health human capital. All those beneficiaries scoring above cut-off points will be automatically invited to access the intervention, and anyone suspected at risk will be offered support and referral to health services.

##### Intervention

The study will pilot a stepped-care mental health program that aims to be structured, brief and includes evidence-based components. The digital intervention will not be developed by the research team. Instead, we will use a third-party platform (SilverCloud®) a virtual mental health platform with eight modules with different tools for comorbid depressive and anxiety symptoms. This platform has been adapted for use by student populations that mainly attend private universities in Colombia and has proven to be effective (25).

**Figure 2:**
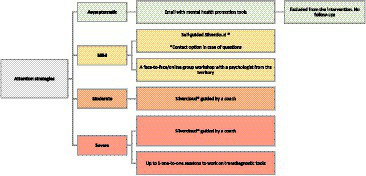
Flow chart of strategies offer within the stepped-care mental health program intervention sites to four levels of severity (asymptomatic, mild, moderate, and severe).

This program is designed to facilitate the participants’ social and labor insertion. The intervention will target depression, anxiety, PTSD, and comorbidities. Participants from the intervention sites will be classified in four levels of severity (asymptomatic, mild, moderate, and severe) according to their scores in these three dimensions encompassing access to a mental-health digital platform and individualized care. They will be offered differential attention strategies (see Figure 2). We will use a transdiagnostic stepped-care intervention program, in which three levels of attention will be in place to respond to the severity of the symptoms as determined by three screening questionnaires encompassing access to a mental-health digital platform and individualized care. This strategy has been planned to provide individualized care for those in severe conditions and considering the high degree of comorbidity documented in conflict zones. The protocol also considers the number of sessions we anticipate will be amenable to our participants. Participants with moderate and mild symptoms will also have access to the digital platform. People with moderate symptoms will have a coach to monitor their progress while the platform is available; people with mild conditions will only be offered the platform. Unlike previous mental health initiatives, we intend to ascertain how to foster the acceptability of a digital strategy, in our initial co-design phase, and the acceptability of personalized virtual sessions with our psychologists.

Asymptomatic participants will receive an email with online resources and tools to help them manage their mental health, and they will not be included as part of the study. The online resources are digital health and psychoeducation platforms that are available nationally and are part of the social outreach initiatives of the Fundación Santa Fe de Bogota. Participants with mild symptoms will receive access to SilverCloud®, a virtual mental health platform with eight modules with different tools for comorbid depressive and anxiety symptoms (26). This platform has been adapted for use by student populations that mainly attend private universities in Colombia, and has proven to be effective (25). The access to the web-based platform will be self-guided. They will also receive an invitation to participate in a mental health workshop in their territories.

For participants with moderate symptoms, the platform will also be available, but in addition, asynchronous feedback will be provided by trained guides (27). The asynchronous feedback will consist of motivational messages as the participants progress through the different modules. The platform also offers participants the opportunity to send messages to the guide and for the guide to respond. Those with severe symptoms will also access SilverCloud® with asynchronous feedback and receive a person-centered intervention delivered by trained psychologists face-to-face or virtually, depending on the participant’s choice.

A group of local psychologists will be trained on different evidence-based tools for depression and anxiety. The training will be done through virtual sessions, and they will receive a manual (developed by the research team on Phase 2) with a step-by-step guide on the intervention. A pre-pilot of the intervention will be done with 10–15 participants with severe scores to supervise adherence to the manual from the psychologists and adjust the protocol, if needed. The intervention is based on CBT and transdiagnostic techniques. It consists of up to five face-to-face or online sessions in which participants will be offered one introduction session with psychoeducation of depression and anxiety from a CBT approach, and at least two psychological tools depending on their symptoms. These tools will include problem-solving, behavioral activation, strengthening of social support, and reduction of safety behaviors. Psychologists will access the participant’s screening scores and progress in SilverCloud® and choose the tools depending on what they observe in the first session. Participants will also receive a workbook containing infographic material on each tool that may be suggested for use by the participants by the psychologists during individualized sessions. Additionally, psychologists will regularly meet with a clinical psychiatrist to discuss participants’ progress.

This strategy has been planned to provide individualized care for those in severe conditions and considering the high degree of comorbidity documented in conflict zones. The protocol also considers the number of sessions we anticipate will be amenable to our participants. Unlike previous mental health initiatives, we intend to ascertain how to foster the acceptability of a digital strategy, and the acceptability of personalized virtual sessions with psychologists.

Participants from the control groups will not receive the intervention until the measures are completed. Afterwards, they will receive access to SilverCloud® (self-guided) and an email with mental health promotion tools. Those who are at risk and need specialized mental health attention (e.g., those with suicidal risk) will be referred to the psychologist of their institutions.

The intervention lasts 16 weeks (4 months), approximately. Phase 3 also includes: one month of recruitment and meetings with the universities and staff from each territory, baseline interview, intervention, endline interview (immediately after the intervention, open for a couple weeks while we reach the target number of participants), follow up (3 months after the deadline), data analysis and presentation of results to the territories.

### Study design, sample, and recruitment

#### Sample size

As a pilot study with no pre-specified effect size hypothesis, it is not relevant to estimate a sample size for hypothesis testing. However, to carry out preliminary analysis of impact, we defined the sample size as follows: assuming an intraclass correlation coefficient of 0.04, an effect size of 0.125 (index f) equivalent to Cohen’s d = 0.25, with a significance level of 5% and a statistical power of 80%, the minimum sample required would be 501 participants (167 per site, of three locations). Our goal is to recruit a minimum of 170 participants from each site, given an expected moderately high attrition rate.

#### Recruitment

Participants in Phases 1 and 2 will be invited via email or in person by JeA program leaders and facilitators. During Phase 3, participants will be invited via email and text messages. Participation is entirely voluntary and will not affect participation in the JeA program. It will be possible for participants to contact researchers directly for further information should they wish not to inform JeA staff of their participation.

#### Inclusion criteria

For all parts of the study, participants must be adults aged 18–28 and registered with the public social program (JeA) living in seven Colombian municipalities affected by the armed conflict. These 7 municipalities are part of the 170 municipalities categorized as “The Development Programs with a Territorial Approach” (PDET in Spanish) by the Havana peace agreement to prioritize social and economic development programs and are the areas most hardly hit by the armed conflict. JeA users are characterized by their low socioeconomic status, according to the National Social Services’ criteria. Many of them are victims of the armed conflict as defined by the victims’ law in Colombia. Participants who meet the inclusion criteria and present anxiety, depression, or post-traumatic stress disorder symptoms will be invited to participate in Phase 3 – piloting the mental health intervention.

#### Study design

Our study is based on a cross-over design, whereby participants in three treatment municipalities (Florencia, Santa Marta, and Valledupar) will be offered the intervention, while participants in four control municipalities (Apartadó, Buenaventura, Ciénega, and San Andrés de Tumaco) will serve as control. Due to ethical reasons, we must offer the intervention to beneficiaries in the control municipalities following the period of intervention in the treatment group. Control municipalities will thus receive the intervention once the two post-treatment measures are taken.

#### Control municipalities

Control municipalities were selected because they share common characteristics with the intervention municipalities in terms of levels of poverty, armed conflict, health, living conditions, public service availability, number of internally displaced people per 100,000, demography, and ethnicity. Additionally, they all have received the “skills for life” training. Control municipalities are also covered by JeA and are PDET territories (conflict-affected areas). To arrive at the control municipalities a proximity score was calculated using the variables with correlations no greater than 0.3 following their standardization (0–1) of their normalized distances.

#### Exclusion criteria

At each phase, if suicidal risk is identified, participants will be referred to the mental health services of their educational institutions or other mental health services in their municipality of residence. They will also be offered access to SilverCloud®. In case of immediate risk to self or others, the consent form will clearly state that we may need to inform the emergency contact participants provide.

#### Informed consent and withdrawal

Informed consent will be on the opening page of the online survey. Participants volunteering to participate in interviews and group discussions will also receive the consent form stating that the conversations will be audio recorded. The consent form will clearly explain what participation entails, that it is possible to withdraw from the study, and that participants can request that their own data be removed from the study until the time the analysis is conducted. A final withdrawal date will also be provided in the information sheet, after which participants may no longer withdraw their data from the study.

#### Data collection

Quantitative data will be collected through an online survey hosted in Redcap. Data collection will be conducted online. The qualitative and co-design elements of the study will be carried out with beneficiaries and staff of the JeA program via video-conferencing software (Teams).

### Screening questionnaire

To better understand young people’s mental health needs and priorities, the resources available, and any contextual factors relevant to the successful implementation a mental health intervention, we will conduct a brief and focused survey of approximately 150 participants (50 per territory). This survey will use the same screening questionnaires we envisage for the intervention to further test their acceptability with this population. JeA will send the questionnaire via email and text messages and invite participants through phone calls. Beneficiaries will be asked what they think about the survey and how it can be improved. We will make changes according to their feedback. This survey will be administered before the intervention and in two follow-ups (immediately after the intervention and three months after).

### Questionnaire instruments

We will invite all students across the selected PDET municipalities to complete the mental health and human capital survey. The following tools will be used to determine the severity of mental health symptoms and human capital outcomes. (1) *Patient Health Questionnaire – PHQ-9*, which is widely used to assess depression symptoms (28), has been validated for use in Colombia (29, 30) and with low income, limited education populations in rural areas with good results (31, 32). Its internal consistency is 0.80. (2) Anxiety will be assessed with GAD-7 (33). Its internal consistency is 0.90, sensitivity 0.92, and specificity 0.83. (3) A Post-traumatic stress disorder brief screen will also be included PC-PTSD-5 (34). It has a test–retest reliability of 0.83, sensitivity with a score of 3 has a sensitivity of 0.93 m specificity is 0.94. (4) Two questions from the Insomnia Severity Index (35, 36), with a reliability of 0,86, sensitivity of 0.87. (5) AF5 Self-Concept Questionnaire (6) Rosenberg Self-esteem scale (RSE) (37). These questionnaires are widely used by studies where the efficacy of interventions that use digital platforms are examined.

In terms of short-term/immediate assessment of the potential of the intervention to improve human capital, we will look at: school attendance (using administrative data on academic enrolment reports), educational permanence (using administrative data on academic permanence reports, every six months), and academic performance (using administrative data on academic excellence report, every six months). Based on data directly collected as part of the pilot intervention, we will also assess the short-term efficacy of the number of hours spent on average during the past academic period studying weekly, the number of hours that beneficiaries dedicate in this academic period to study weekly and the self-reported grade point average in current and past academic periods. In terms of the longer-term efficacy of the intervention, we will look at: certification (whether completed educational program, based on JeA administrative records) and formal labor market inclusion (whether working in a formal job, based on PILA Administrative Registry). Furthermore, we will also assess the efficacy of the mental health intervention on self-esteem (RSES) and self-concept (AF5).

**Figure 3:**
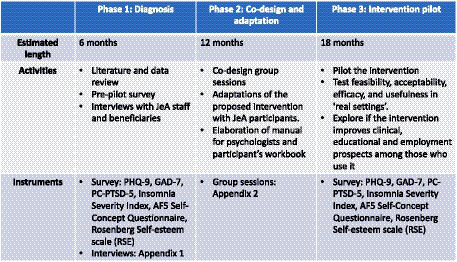
Summary of pilot study phases.

Figure 3 summarizes the estimated length, activities, and instruments used in each of the phases.

## Data analysis

### Assessing feasibility and acceptability

Feasibility, acceptability, and potential efficacy will be assessed using a “hybrid effectiveness-implementation” approach (38). We will use standard measures across all study sites to facilitate within and cross-site comparisons in efficacy. We will interview a subsample of 5–6 participants in each municipality at month three to explore acceptability qualitatively and learn if different municipalities need different implementation strategies and possible reasons why the intervention failed or succeeded.

### Quantitative analysis of impact

Our primary aim will be to assess the impact of the intervention on our mental health and human capital outcomes. We will use regression analysis with a difference-in-differences design, which compares changes in outcomes before and after intervention measures across participants in treatment and control areas. This approach considers human capital as an outcome embodied in individuals by exploring the variation in individual knowledge (cognitive skills captured by academic performance) and individual abilities (non-cognitive skills acquired as life competencies). Thus, we will look at the magnitude of mental health-based rehabilitation on young adult students across different dimensions of mental health (depression and anxiety, fundamentally), which is considered beneficial due to human capital accumulation.

Since individual observations will be collected in different regions, we will adjust for clustered data. In the absence of treatment randomization that assures that treatment and control groups are exchangeable, this quasi-experimental design helps us to control for time-invariant differences between treatment and control areas by focusing on changes, rather than levels, in outcomes. To address bias from time-varying confounders, we will incorporate controls for time-varying factors in all analyses. We will all report the prevalence of depression, anxiety, and PTSD, along with their commodities and the effectiveness outcome, which will be the proportion of participants with a reduction in their baseline symptoms. To determine the changes in human capital, we will use both administrative and survey data that measure continued JeA program enrolment, accumulated grade point average, and self-reported hours dedicated to study. This will be carried out in STATA-19.

### Qualitative analysis

Interviews will be transcribed verbatim (in Spanish) and analyzed thematically using the Clarke Braun and Clarke (39) approach. Analysis will be partly deductive, seeking to identify emotional ‘touchpoints’ (40) and other salient aspects of participants’ experiences of seeking or providing support for mental and emotional distress to inform and shape the planned intervention, and partly inductive, exploring themes identified as significant during the analytical process itself. The analysis will follow the 6 steps Clarke Braun and Clarke (39) recommended: 1. Familiarization with participants’ responses. 2. Generation of initial codes based on both deductive and inductive approach; 3. Identification of themes; 4. Review of themes and further coding; 5. Refinement of themes; 6. Writing to illustrate the analytical themes, relevance, and study implications (39). The analysis will be carried out by one researcher on the team, with regular discussion with and review by another member of the team with qualitative expertise. Data will be analyzed as is generated, with themes from the interviews informing the questions for the group discussions. The latter will be held online via Microsoft Teams and facilitated by two members of the research team. Segments of the recordings with direct relevance to the ongoing analysis and to the development and refinement of the planned intervention will be transcribed to contribute to the analysis. The finalized analysis will be translated into English for publication purposes.

### Ethics and data management

This study was approved by the Ethics Committees of Fundación Santa Fe de Bogota (CCEI-13269-2021) and King’s College London (HR/DP-21/22-22947). We have established a data management protocol, and procedures for team communications, data storage, security, and backup. All scales used are available in the public domain.

One-to-one interviews will be transcribed by a research team member or by professional transcribers providing a suitable formal confidentiality agreement as per our data management plan. Full confidentiality cannot be guaranteed for focus groups and co-design group work. Still, we will ensure that participants know this and commit to avoiding sharing the information discussed in the groups outside of the sessions. Qualitative and quantitative data will be deidentified when writing the results.

Participating JeA staff and beneficiaries will be recruited with the help of JeA staff. It is possible that, where power differentials are involved, some participants may feel obliged to participate in the study (perceived coercion) if invited by senior managers and/or course tutors. To mitigate this possibility, we will ensure that all the study information clearly indicates that participation is voluntary and that declining to participate has no financial, professional, academic, or other implications. No identified data will be shared with JeA.

## Discussion

There is some evidence that mental health interventions can help the millions of vulnerable populations affected by conflict, but challenges in their implementation remain (13, 14). JeA is a major national program of the Colombian government to decrease economic disparities by promoting post-secondary education among vulnerable young people from low-income backgrounds. Yet, participants in the program face major mental health problems and lack adequate access to treatment and support. Our project aims to test the impact of a stepped-care mental health intervention that offers digital and professional mental health support to students with symptoms of depression, anxiety or PTSD who live in conflict-affected areas in Colombia. The study will examine the feasibility and acceptability of the intervention, and it will assess its effectiveness to improve the mental health and human capital outcomes of beneficiaries.

Findings from this study will help identify strategies to address mental health problems among socioeconomically vulnerable young people that can be adapted to different contexts in in low and middle-income countries. Our study will help to address a research gap on the implementation and efficacy of transdiagnostic stepped-care interventions that incorporate a digital mental health component for young people, an important gap highlighted in recent reports (14). Through collaboration with the Government department responsible for implementation, we also aim to provide evidence that will potentially enable long-term adoption of the intervention in the Youth in Action program. Our findings will have an important impact for victims of the conflict, whose mental health is now established as a priority in law, though evidence of the effectiveness of interventions is lacking. Should a digital mental health intervention be shown to be both feasible and effective, policies may support digital interventions as a way to improve access for people displaced by the conflict and struggling to access in person treatment.

## Ethics statement

The studies involving humans were approved by Ethics Committees of Fundación Santa Fe de Bogota (CCEI-13269-2021) and King’s College London (HR/DP-21/22-22947). The studies were conducted in accordance with the local legislation and institutional requirements. The participants provided their written informed consent to participate in this study. Written informed consent was obtained from the individual(s) for the publication of any potentially identifiable images or data included in this article.

## Author contributions

AZ, MG, FI, RA, MA, and SD wrote the main manuscript text. MG, FI, and MA prepared Figures 1, 2. All authors reviewed the manuscript and approved the submitted version.
