# Supplementary material for: Piloting a mental health intervention for young adults in poverty enrolled in post-secondary education in post-conflict regions in Colombia: a study protocol
Source: Front Psychiatry. 2023 Nov 13;14:1238725. doi: 10.3389/fpsyt.2023.1238725 (PMC10686213; doi:10.3389/fpsyt.2023.1238725)
Supplement: Supplementary file 1 [file Data_Sheet_1.docx]

Appendix 1

Youth in Action (JeA) Beneficiaries’ Guide

1. Summarise project and confirm consent/address any questions. Make it especially clear that they can decline to answer any of the questions.
2. Can you tell me something about yourself? (Let the participant introduce him/herself, state his/her age, place of residence, and program of study). How long were you part/ have been part of the Youth in Action program? Are you happy with the program? In what areas of your life do you think being part of Youth in Action has helped you?
3. Through various investigations, we know that many people your age in Colombia have experienced emotional and mental distress. Have you ever seen this in some of your friends?  [explore circumstances sensitively – do not ask for details of the distress, only about the support they received if any]
4. In your experience, what do young people do when they experience this type of feelings?
5. Do you think that young people, when they experience this type of feelings (anguish, sadness seek help or advice? (Explore which type of help and factors that influence support-seeking behaviours)
6. What does mental health mean to you?
7. If you or any of your friends participating in Youth in Action needed mental health support, where could you seek help?
8. Do you think this kind of help exists at Youth in Action?
9. Do you think these services are effective?
10. Have you or your friends ever had access to these services?
11. Have you participated in the Skills for Life training? Do you consider that your participation in Skills for Life has given you tools to address situations related to mental health?
12. What do young people mention are the main barriers to getting mental health help/support?
13. What do young people say they find helpful in seeking support?
14. Do you think the pandemic affected how young people in Colombia can seek and access mental health support?
15. If you had a magic wand, what are the top two things you would change or implement to support young people struggling with their mental and emotional health?
16. Do you think what you mention would be different in conflict-affected areas?
17. Is there anything you want to add that we haven't asked you about?

Youth in Action (JeA) Staff Guide

1. Summarise project and confirm consent/address any questions.
2. Can you tell me a little bit about your role in JeA and how long you’ve been part of this initiative?
3. In your experience of working with young people in the context of the JeA programme, have you come across young people suffering from mental and/or emotional distress? [explore frequency, severity, distribution, possible correlations, role of conflict and situation in conflict-affected areas if known etc]
4. Do you have experience of supporting young people with mental and/or emotional distress?

If yes, explore: what are the main challenges to providing support?

What do young people mention as the main obstacles to obtaining support?

What do young people say they find helpful in seeking support?

Are there any specific needs for young people from conflict-affected areas?

1. If not to (4) >>> In your experience of supporting young people in developing life and learning skills, what are the main challenges to engaging young people in the programme?

Explore further: what do young people mention as the main obstacles to accessing the programme?

What do young people say they find useful about the programme?

Are there any specific needs or circumstances for young people from conflict-affected areas?

1. Do you think the pandemic affected the ways in which young people in Colombia can seek and access support? How?
2. If you had a magic wand, what are two main things you’d change or introduce to support young people who struggle with their mental and emotional health?
3. Would that be any different in conflict-affected areas?
4. Is there anything you might want to add, that I did not ask you about?
