# Supplementary material for: Piloting a mental health intervention for young adults in poverty enrolled in post-secondary education in post-conflict regions in Colombia: a study protocol
Source: Front Psychiatry. 2023 Nov 13;14:1238725. doi: 10.3389/fpsyt.2023.1238725 (PMC10686213; doi:10.3389/fpsyt.2023.1238725)
Supplement: Supplementary file 2 [file Data_Sheet_2.docx]

Appendix 2

Focus Group 1 - Youth in Action staff

Five main thematic blocks:

1. How to identify problems early and recognize signs to prevent mental health problems rather than treating them?

2. How to raise awareness of mental health problems, not only at the individual level, but also at the family and social level?

3. The type of language that should be used in the intervention. How the intervention should address mental health issues. How stigma relates to language.

4. Life skills module: Explore if the module could be a gateway to deepen the topic of mental health, for example, including workshops and talks accompanied by dissemination campaigns through social networks that allow beneficiaries to know about the service and how they can access it. Understand if the communication campaign of our service should be through the module, through social media, or both.

5. Should the intervention be different for conflict-affected and non-affected youth?

Focus Group 2 - Youth in Action beneficiaries

Five main thematic blocks

1. The taboo around mental health and seeking help. How to prevent stigma around mental health.

2. How to use language to address stigma and increase participation in services.

3. How to recognize early signs or symptoms of mental illness in young people or their peers.

4. Do's and don'ts on a virtual platform. How they feel about SilverCloud.

5. Targeted interventions for conflict-affected youth.

Focus Group 3 - Youth in Action beneficiaries and staff

Four main blocks

1. Language: Should we use the term mental health or change it to wellness?

2. How to address the priorities that emerged from the beneficiary and staff interviews and focus groups.

3. What we can and can't adapt in SilverCloud. How to make the things we can't customize work.

4. Differentiated approach for victims of armed conflict
